# Phospho-mimetic CheV interacts with a subset of chemoreceptors

**DOI:** 10.1128/mbio.02874-25

**Published:** 2025-11-10

**Authors:** Miguel A. Matilla, Mario Cano-Muñoz, Elizabet Monteagudo-Cascales, Tino Krell

**Affiliations:** 1 Department of Biotechnology and Environmental Protection, Estación Experimental del Zaidín, Consejo Superior de Investigaciones Científicas, Granada, Spain; Massachusetts Institute of Technology, Cambridge, Massachusetts, USA

**Keywords:** chemotaxis, chemoreceptor, *Pseudomonas aeruginosa*, CheV

## Abstract

Chemotaxis pathways are among the most complex signaling systems in bacteria. A central feature of these pathways is the ternary complex formed by chemoreceptors, the autokinase CheA, and the coupling proteins CheW and CheV. Whereas CheW is present in all chemotaxis pathways, CheV is primarily found in bacteria that contain many chemoreceptors. CheV is a fusion of a CheW-like domain to a phosphorylatable receiver domain. The roles of CheV and its phosphorylation are currently uncertain. *Pseudomonas aeruginosa* contains many chemoreceptors for which the cognate signals have been identified. Quantitative capillary chemotaxis assays of a *cheV* mutant revealed that responses to certain chemoeffectors, such as nitrate and α-ketoglutarate, were drastically reduced, while responses to others, such as amino acids and inorganic phosphate, were comparable to the wild type, indicating that CheV selectively acts on specific chemoreceptors. To study the mechanism of CheV action, we conducted protein-protein interaction experiments using isothermal titration calorimetry. These studies showed that unphosphorylated CheV fails to bind to cytosolic fragments of the McpN and PctA chemoreceptors, which mediate responses to nitrate and amino acids, respectively. In contrast, the phosphorylation-mimic CheV D238E bound with very high affinity (*K*_D_ = 8 nM) to McpN but failed to interact with PctA. Thus, CheV in *P. aeruginosa* binds to some chemoreceptors but not to others in a phosphorylation-dependent manner. These results suggest that CheV is a regulatory protein that modulates signaling through specific chemoreceptors. CheV may thus facilitate the coordination of chemotaxis responses in complex, multi-chemoreceptor systems.

**IMPORTANCE:**

CheV is one of the least understood chemosensory signaling proteins. Our demonstration that CheV interacts only with certain chemoreceptors offers fundamental new insights. These findings, combined with the observation that CheV is present in bacteria with numerous chemoreceptors, suggest that CheV plays a role in coordinating chemotactic outputs in complex chemosensory systems. Understanding the mechanisms by which chemotactic responses are defined in bacteria with a high number of chemoreceptors is a major research priority in the field of chemotaxis. While previous studies, including this one, show that the ability to be phosphorylated is crucial for CheV function, the molecular consequences of CheV phosphorylation have remained unclear. Our discovery that phosphorylation is essential for CheV binding to certain chemoreceptors fills in this critical gap in understanding the molecular mechanism of CheV. This study is likely to inspire further research into CheV function in other bacteria using similar approaches.

## INTRODUCTION

Chemosensory pathways are among the most sophisticated signal transduction systems in bacteria (1, 2). Genome analyses reveal that more than half of the sequenced bacterial genomes encode chemosensory signaling proteins (3). While most chemosensory pathways mediate chemotaxis, others carry out alternative cellular functions, like the control of second messenger levels, or are involved in twitching motility and mechanosensing (3–5).

The core of the chemosensory pathway is formed by a ternary complex comprised of chemoreceptors, the CheA autokinase, and a coupling protein. Signaling is typically initiated by ligand binding to the chemoreceptor ligand-binding domain (LBD), which in turn modulates the activity of CheA and the subsequent transfer of the phosphoryl group to the CheY response regulator. Other essential components of the pathway are the CheR methyltransferase and the CheB methylesterase, whose coordinate activities control the methylation state of the chemoreceptors (1, 2).

Importantly, there are two coupling proteins in some bacteria, CheW and CheV (6, 7). CheW consists of a single domain and is essential for the formation of hexagonally arranged chemosensory arrays (8). In contrast, CheV is a fusion of a CheW-like domain with a phosphorylatable receiver domain. All bacterial chemosensory pathways contain either CheW, CheV, or both (7, 9). Several studies revealed a partial functional redundancy of CheW and CheV. Single deletions of the *cheV* or *cheW* genes in *Bacillus subtilis* (10) and *Campylobacter jejuni* (11) cause either no or only minor reductions in chemotaxis. However, the *cheV*/*cheW* double mutant of *B. subtilis* is completely defective for chemotaxis (10). In contrast, in *Helicobacter pylori,* CheW and CheV_1_ were non-redundant since mutants in *cheW* or *cheV1* are unable to activate the CheA kinase (12, 13). This non-redundancy is further supported by the finding that *H. pylori* mutants deficient in either *cheW* or *cheV*_1_ fail to form the chemosensory array (14). Studies with other bacteria found a significant reduction, but not an absence of chemotaxis in *cheV* and *cheW* single mutants (15–17). The phosphorylation of CheV is required for its activity (18). Experimental data and comparative genomic analyses support the idea that CheV acts as a phosphate sink (12, 18, 19). The concept of a phosphate sink is rooted in the generally faster hydrolysis of phospho-aspartate, present in CheY, CheB, and CheV, compared to phospho-histidine, as found in CheA (20, 21). CheV would enhance the dephosphorylation of CheA, subsequently influencing the concentration of phosphorylated CheY.

Bacterial adenylate cyclase two-hybrid (BACTH) assays demonstrated that the signaling domains of the Tlp1 (11) and Tlp10 (22) chemoreceptor of *C. jejuni* interact stronger with CheV than CheW, whereas both proteins interacted with comparable strength with the Tlp2, Tlp3, Tlp4 (23), and Tlp11 chemoreceptor signaling domains (24). However, this preferential interaction of CheV was not reflected in the magnitude of chemotaxis since the Tlp1-mediated responses of the *cheV* and *cheW* mutants were comparable and similar to the wild type (wt) (11).

CheV is among the least understood chemosensory signaling proteins (3). Key open questions include the physiological advantages of having two coupling proteins in the ternary chemotactic signaling complex, the functional implications of CheV phosphorylation, and why *cheV* genes are predominantly found in bacteria with numerous chemoreceptors (19). An interesting insight was provided by a bioinformatic analysis of *cheV* genes in enterobacteria (19). Evolutionary analyses showed that CheV co-evolved with a specific family of chemoreceptors (19), suggesting that CheV may contribute differentially to chemotaxis mediated by different chemoreceptors. However, experimental data to support this hypothesis are lacking.

**Fig 1:**
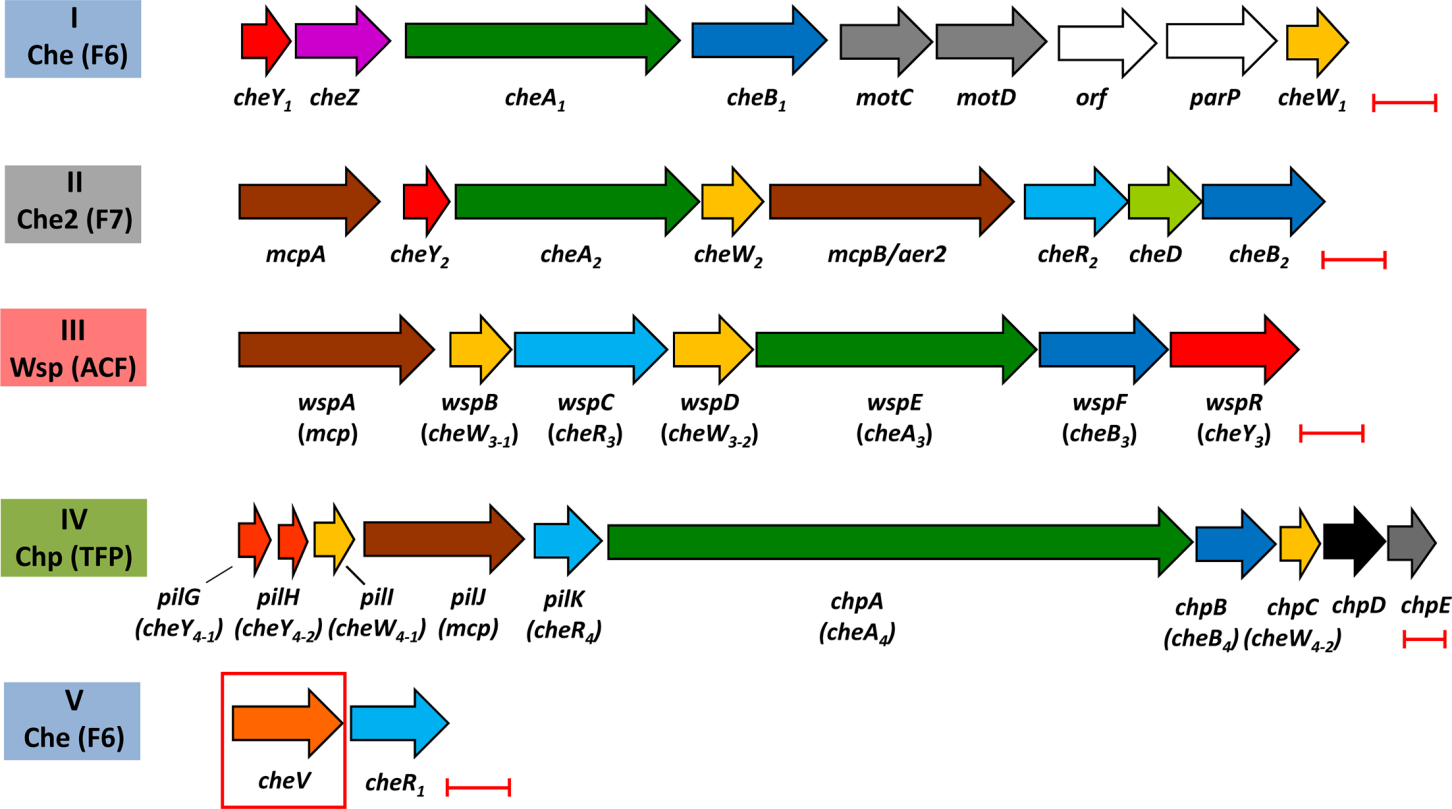
The five gene clusters that encode the signaling proteins of the four chemosensory pathways in *Pseudomonas aeruginosa* PAO1. Clusters I and V encode the proteins of the Che pathway (chemotaxis), cluster II encodes the Che2 pathway proteins (virulence by unknown mechanisms), cluster III corresponds to the Wsp pathway (c-di-GMP homeostasis), and cluster IV encodes the Chp pathway (twitching and mechanosensing). Pathway classification according to (3) is shown. Wsp: wrinkly spreader phenotype; Chp: chemosensory pili; F: flagellar motility; ACF: alternative cellular functions; TFP: type IV pili. Scale bars: 0.5 kbp.

To address this question, we conducted quantitative chemotaxis experiments with *Pseudomonas aeruginosa* PAO1, a model strain in the study of chemotaxis (25). *P. aeruginosa* is among the most important human pathogens; it kills about half a million people annually (26, 27). PAO1 has five gene clusters that encode chemosensory signaling proteins. These function in four distinct chemosensory pathways (Fig. 1) (28). The Che pathway mediates chemotaxis, the Che2 pathway plays a role in virulence by an unknown mechanism, the Wsp pathway regulates c-di-GMP levels, and the Chp system is involved in twitching and mechanosensing (5, 28–30). Signaling through all four pathways is important for efficient virulence (28).

Strain PAO1 has 26 chemoreceptors. Bioinformatic and experimental data suggest that 23 receptors stimulate the Che pathway, whereas each of the remaining three chemoreceptors feeds into either the Che2, Wsp, or Chp pathway (31). PAO1 has a single CheV that is encoded in chemotaxis gene cluster V (Fig. 1). The signals recognized by about half of the PAO1 chemoreceptors have been identified (32). In most cases, mutants defective in these chemoreceptors showed either a complete loss or a large reduction in the chemotactic responses to their cognate ligands, indicating that the corresponding wt responses are primarily due to the action of a single chemoreceptor (see Table 1). This, and the wealth of information available on the function of its chemoreceptors, makes PAO1 a valuable system well-suited to assess the contribution of CheV to the responses mediated by specific receptors.

**TABLE 1:** Summary of data available on chemoreceptors that mediate chemotactic responses to different chemoeffectors in *P. aeruginosa* PAO1^*a*^

Chemoeffector	Receptor	Binding mode/*K*_D_ (References^***b***^)	Signaling domain family^*c*^	Receptor LBD family	References^*d*^	Chemotaxis in *cheV* mutant relative to wt (%)
NaNO_3_	**McpN**	**direct (47 µM**) (33)	**40H**	**PilJ**	(33)	**5**
L-malate	CtpM	direct (23 µM) (34)	40H	sCache_2	(34, 35)	92
Histamine	**TlpQ**	**direct (639 nM**) (36)	**40H**	**dCache^*e*^**	(36)	**51**
α-ketoglutarate	**McpK**	**direct (301/81 µM)^*f*^** (37)	**40H**	**HBM**	(37)	**23**
Inorganic phosphate	CtpL	indirect (3.7 µM)^*g*^ (38)	40H	HBM^*e*^	(38, 39)	39
	CtpH	direct (22 µM) (38)	40H	4HB^*e*^		94
L-Val	PctA	direct (3 µM) (40)	40H	dCache_1	(40–42)	83
L-Gln	PctB	direct (1.2 µM) (40)	40H	dCache_1	(40–42)	100
γ-aminobutyrate	PctC	direct (1.2 µM) (40)	40H	dCache_1	(40, 41, 43)	101
Acetylcholine	**PctD**	**direct (23 µM**) (44)	**40H**	**dCache^*e*^**	(44)	**45**
Oxygen	**Aer/PA1561**	**direct^*h*^**	**40H**	**PAS**	(45)	**Not detectable**

^*a*^ Responses of the wt strains and the *cheV* mutant are shown in Fig. 3. LBD families are defined according to Pfam (46) and, in the absence of annotation, by inspection of the AlphaFold2 (47) model. Tactic responses that were significantly different in the *cheV* mutant compared to the wt strain are shown in bold.

^*b*^ reference for dissociation constant.

^*c*^ according to (9, 48).

^*d*^ references that document the functional relationship between chemoreceptor and chemoeffector.

^*e*^ Inspection of an AlphaFold2 model.

^*f*^ binding with positive cooperativity.

^*g*^ binding of Pi-loaded PstS to the CtpL-LBD.

^*h*^ by homology with *E. coli* Aer.

We show that CheV participates in the signaling of only a subset of chemoreceptors, highlighting its role in regulating complex responses in systems with many chemoreceptors. We also demonstrate the functional relevance of CheV phosphorylation by showing that only a CheV phospho-mimic, but not unphosphorylated CheV, binds to chemoreceptors. Our study provides novel insight into the physiological function of one of the least understood chemosensory signaling proteins. It should provide an impetus to explore the role of CheV in other bacteria.

## RESULTS

### CheW_1_ of *P. aeruginosa* is required for chemotaxis

**Fig 2:**
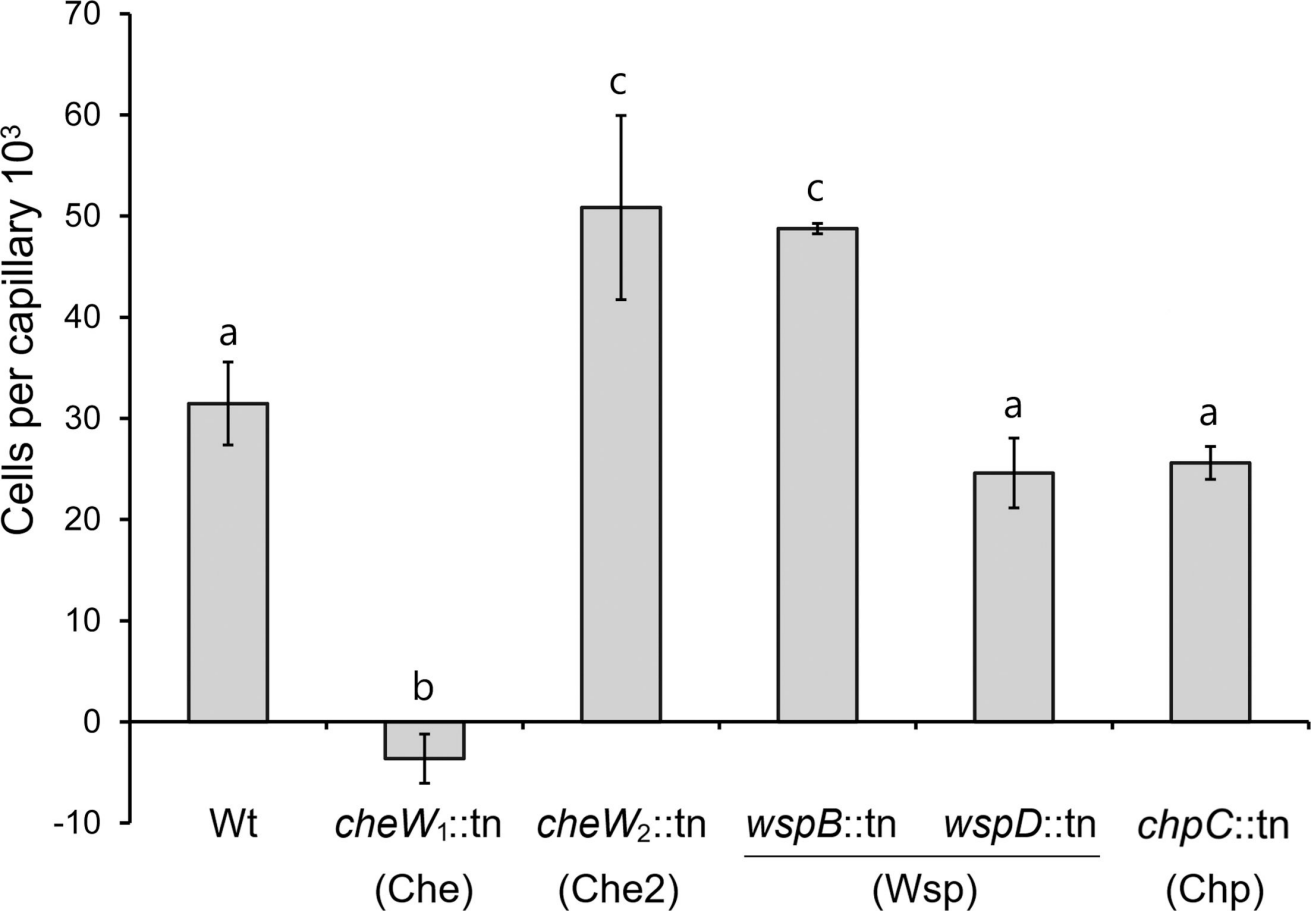
Specificity of interaction of CheW_1_ with the Che pathway. Quantitative capillary chemotaxis assays of *P. aeruginosa* PAO1 and five *cheW* mutants. The attractant was 1 mM L-cysteine. Data were corrected for the number of bacteria that swam into buffer-containing capillaries (14,400 ± 901 for wt; 13,800 ± 3,400 for *cheW*_1_::tn; 18,444 ± 4,986 for *cheW*_2_::tn; 17,333 ± 2,142 for *wspB*::tn; 16,222 ± 4,384 for *wspD*::tn; 13,200 ± 1,047 for *chpC*::tn). The corresponding chemosensory pathways are indicated in brackets. Bars with the same letter are not significantly different (*P*-value < 0.05; by Student’s *t*-test).

*P. aeruginosa* PAO1 has six CheW homologs (Fig. 1) (9). Gene clusters III (Wsp pathway) and IV (Chp pathway) each harbor two *cheW* genes, whereas there is a single *cheW* gene in clusters I (Che pathway) and II (Che2 pathway). These CheW proteins share a modest sequence identity of 14 to 30% (Fig. S1). We assessed the role of five CheW homologs associated with the four chemosensory pathways: CheW_1_, CheW_2_, WspB, WspD, and ChpC, and conducted quantitative capillary chemotaxis assays with the wt strain and single mutants in each of the respective genes encoding CheW homologs to 1 mM L-cysteine, which triggers a strong chemoattractant response in strain PAO1. We observed a lack of chemoattraction in the *cheW*_1_ mutant (Fig. 2), which corresponds to the *cheW* homolog in the Che pathway (Fig. 1). The responses of the remaining four mutants were either similar to or superior to those of the wt (Fig. 2). These findings indicate a specific interaction of the CheW encoded in the Che gene cluster with the other proteins of the Che pathway, supporting previous findings that the *P. aeruginosa* chemosensory proteins assemble into insulated pathways (30, 49).

### CheV is required for chemotaxis mediated by a subset of *P. aeruginosa* chemoreceptors

*P. aeruginosa* has one CheV protein, which is encoded in the chemosensory gene cluster V together with the CheR_1_ methyltransferase (Fig. 1) (9, 21, 24). To assess the contribution of CheV to responses mediated by different chemoreceptors, we selected chemoeffectors for which the corresponding chemoreceptor has been identified (Table 1). In most of the cases, single chemoreceptor mutants are unable to mediate chemotaxis to their cognate ligands (Table 1). This permits us to attribute a specific response to individual chemoreceptors.

**Fig 3:**
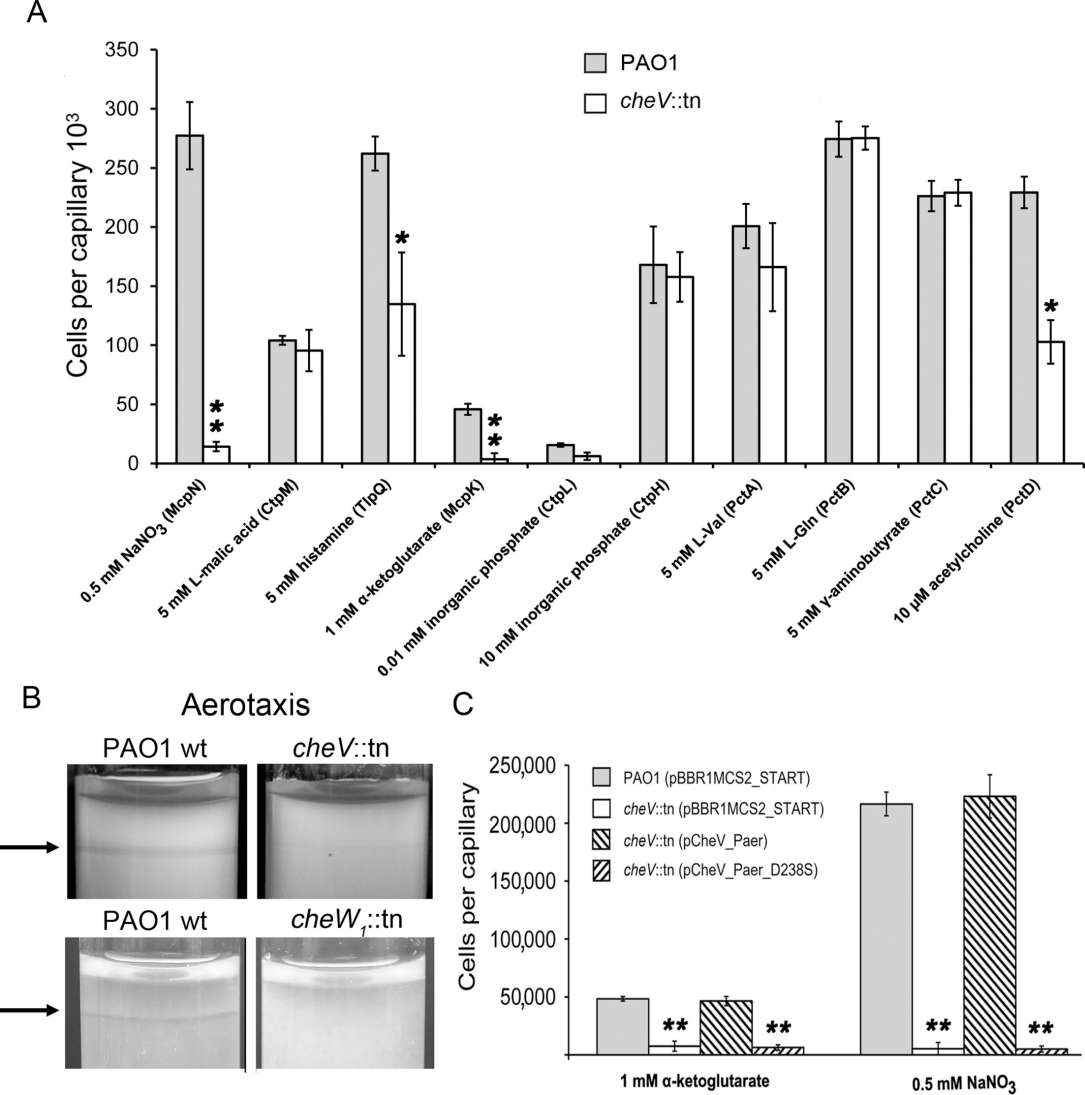
Tactic responses of *P. aeruginosa* and its *cheV* mutant towards different signals. (**A**) Quantitative capillary chemotaxis assays to the indicated compounds. The corresponding chemoreceptors are shown in brackets. Data were corrected for the number of bacteria that swam into buffer-containing capillaries (14,130 ± 2,232 for wt; 11,366 ± 2,221 for *cheV*::tn). Information on the chemoreceptor-chemoeffector interaction is provided in Table 1. (**B**) Aerotaxis tube assays. The arrow points to the band characteristic for an aerotaxis response. (**C**) Quantitative capillary chemotaxis assays to α-ketoglutarate and nitrate of *P. aeruginosa* strains harboring different pBBR-based plasmids. Data were corrected for the number of bacteria that swam into buffer-containing capillaries (8,725 ± 3,344 for PAO1 (pBBR1MCS2_START); 6,208 ± 2,734 for *cheV*::tn (pBBR1MCS2_START); 9,883 ± 3,514 for *cheV*::tn (pCheV_Paer); 8,291 ± 2,227 for *cheV*::tn (pCheV_Paer_D238S). pBBR1MCS2_START: empty plasmid; pCheV_Paer: pBBR1MCS2_START plasmid encoding wt CheV; pCheV_Paer_D238S: pBBR1MCS2_START plasmid encoding the CheV D238S variant, in which the phosphoryl-accepting Asp residue has been replaced by Ser. Student’s *t*-test: *P-value < 0.05, **P-value < 0.01.

Quantitative capillary chemotaxis assays to four chemoeffectors recognized by McpN, McpK, TlpQ, and PctD, respectively, and aerotaxis responses were significantly reduced in the *cheV* mutant as compared to the wt strain (Fig. 3A and B). Chemotaxis to the remaining six chemoeffectors tested was comparable to that of the wt strain (Fig. 3A). Responses to nitrate and α-ketoglutarate were down by ~95 and ~92%, respectively, and chemotaxis to histamine and acetylcholine was reduced by about half in the *cheV* mutant (Fig. 3A). Aerotaxis assays of the wt strain showed the characteristic band for aerotaxis close to the air interface. This band was not obvious with the *cheV* mutant (Fig. 3B), indicating that CheV is important for aerotaxis. The chemoreceptors whose responses were diminished in the *cheV* mutant belong to different families containing PilJ-, dCache-, HBM-, or PAS-type LBDs (Table 1). Furthermore, the aerotaxis receptor Aer contains a cytosolic LBD, whereas the LBDs of the remaining chemoreceptors are located in the periplasm. The signaling domains of CheV-dependent and independent receptors all belong to the 40 H (heptad repeat) family (9, 48).

When *cheV* was expressed *in trans* in the *cheV*-deficient strain, aerotaxis and chemotaxis to α-ketoglutarate, nitrate, histamine, and acetylcholine in the *cheV* mutant were fully restored (Fig. 3C; Fig. S2). UniProt predicts D238 as the phosphorylation site in CheV. We have confirmed this prediction by a structural alignment of the CheV AlphaFold (47) model with the 3D structure of the Spo0A response regulator that was obtained in its phosphorylated state (50) (Fig. S3). When a plasmid encoding CheV D238S was used for complementation, no chemotaxis was observed (Fig. 3C), indicating that phosphorylation was essential for CheV activity. This finding is consistent with previous studies on *B. subtilis* CheV (18). Analyses with the Rosetta Stability and Scoring modeling software (51) indicated that this mutation does not reduce protein stability (Table S1).

### CheV and CheW_1_ are both required for responses mediated by CheV-dependent chemoreceptors

**Fig 4:**
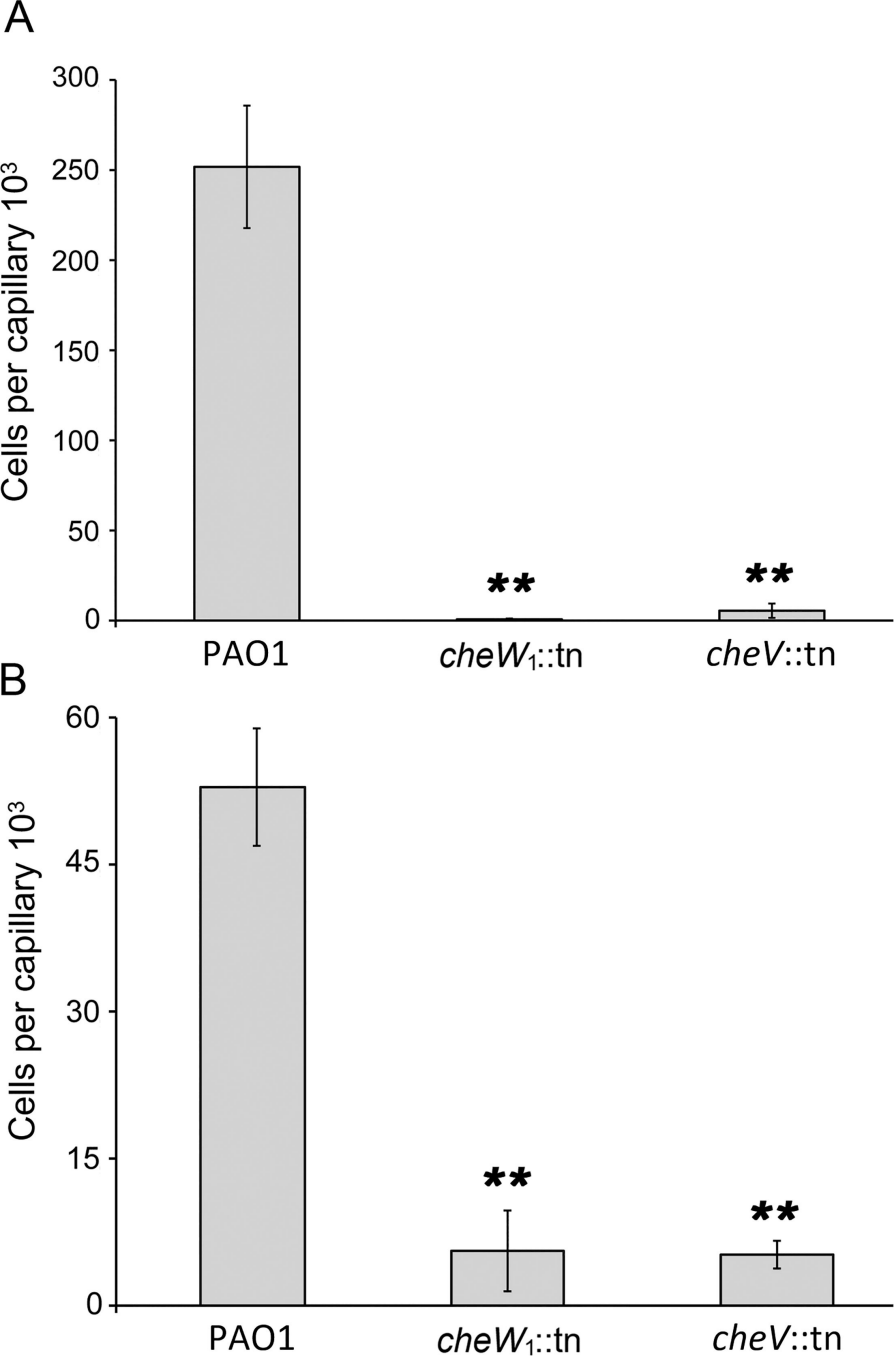
Both CheW_1_ and CheV are required for chemotactic responses of *P. aeruginosa* PAO1 to nitrate and α-ketoglutarate. Quantitative capillary chemotaxis assays of the wt and mutants in *cheW*_1_ or *cheV* to 0.5 mM NaNO_3_ (**A**) or 1 mM α-ketoglutarate (**B**). Data were corrected for the number of bacteria that swam into buffer-containing capillaries (20,227 ± 2,314 for PAO1 wt; 9,911 ± 3,694 for *cheW*_1_::tn; 13,050 ± 4,340 for *cheV*::tn). Student’s *t*-test: ** *P*-value < 0.01.

As shown above, inactivation of *cheV* reduced chemotaxis to nitrate, α-ketoglutarate, histamine, and acetylcholine (Fig. 3A) and abolished aerotaxis (Fig. 3B). To assess the role of CheW_1_ in these responses, we conducted chemotaxis assays with the *cheW*_1_ mutant. We found that the *cheW*_1_ mutant failed to respond to nitrate, α-ketoglutarate, histamine, and acetylcholine (Fig. 4; Fig. S4) and was unable to perform aerotaxis (Fig. 3B). Previous studies in other species have shown that CheV and CheW are either functionally redundant (10, 11) or not (13, 14). Our data show that the latter case applies to *P. aeruginosa* CheW_1_ and CheV.

### A phosphorylation mimic of CheV binds to a cytosolic fragment of McpN but not to a cytosolic fragment of PctA

The *P. aeruginosa cheV* mutant fails to respond to nitrate, whereas responses to different amino acids were comparable to the wt strain (Fig. 3A). Nitrate chemotaxis is mediated by the McpN chemoreceptor (33), whereas amino acid chemotaxis is mediated by the three receptors PctA, PctB, and PctC (41). The latter three receptors are paralogous, and their cytosolic fragments share 93% sequence identity (52). To test whether CheV interacts with McpN but not with PctA, we generated pET-based expression vectors containing the *cheV* gene and the DNA sequences encoding the cytosolic fragments of McpN (McpN_CF) and PctA (PctA_CF). The corresponding proteins were overexpressed in *E. coli* and purified. We then conducted isothermal titration calorimetry (ITC) experiments to study protein–protein interactions.

**Fig 5:**
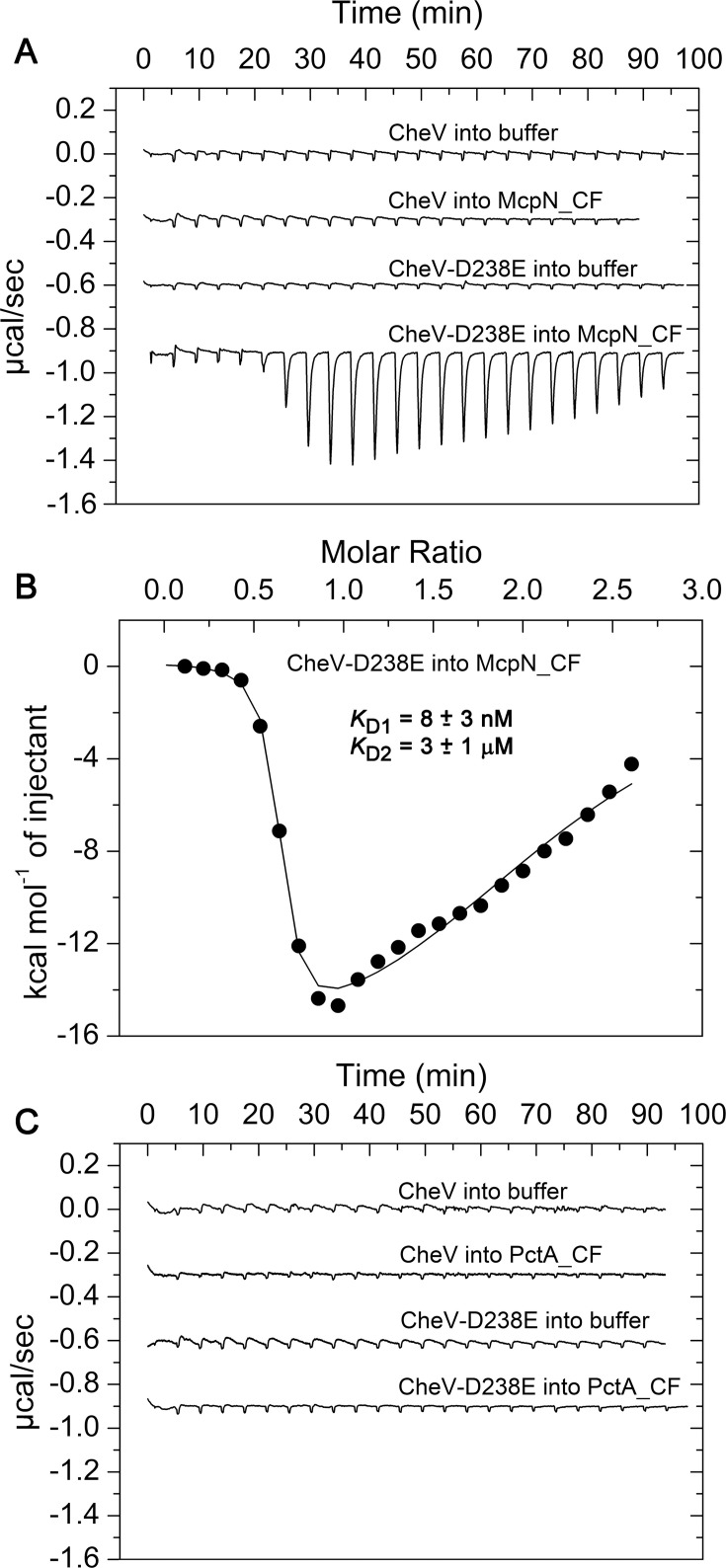
Isothermal titration calorimetry study of the binding of CheV and the phosphorylation mimic CheV D238E to the cytosolic fragments of the McpN and PctA chemoreceptors. Titration of either 10 µM McpN_CF (**A**) or PctA_CF (**C**) with 113 µM CheV or CheV-D238E. A single 1.60 µL injection was followed by a series of 12.82 µL injections. Shown are also the control titration of buffer with CheV and CheV-D238E. (**B**) Concentration-normalized and dilution-heat-corrected titration data for the binding of CheV-D238E to McpN_CF. Data were fitted with the "Two Binding Sites" model in the MicroCal version of Origin software for ITC.

The control titration of buffer with CheV resulted in small and uniform peaks, representing dilution heats (Fig. 5A). A similar curve was obtained when McpN_CF was titrated with unphosphorylated CheV. Since the phospho-Asp bond is labile, no stably phosphorylated receiver domains can be generated for experimentation (53). However, numerous studies show that the replacement of the phospho-accepting aspartate with glutamate mimics protein phosphorylation (54–56). Therefore, we generated the CheV D238E mutant for use in ITC assays. Titration of McpN_CF with CheV D238E resulted in a response in which two binding events could be distinguished: an initial high-affinity endothermic event with a *K*_D_ of 8 ± 3 nM followed by an exothermic event of lower affinity (*K*_D _= 3 ± 1 µM). An n-value of close to 0.5 was obtained for the first event indicative of the binding of a CheV D238E monomer to the McpN_CF dimer. No reliable information on the stoichiometry of interaction can be obtained from hyperbolic traces such as the second exothermic event. We hypothesize that this second event represents binding of another CheV to the opposing face of the McpN_CF dimer (Fig. S5). As shown in Fig. 5C, neither CheV nor CheV D238E bound to PctA_CF. It was shown in *H. pylori* that both CheW and CheV are required to form functional chemosensory arrays. To assess whether *P. aeruginosa* CheW_1_ may be required for the binding of CheV D238E to PctA_CF, we produced CheW_1_ as a recombinant purified protein. We repeated the microcalorimetric titration of PctA_CF with CheV D238E in the presence of saturating concentrations of CheW_1_ (Fig. S6). However, no binding heats were observed, indicating that CheW_1_ does not facilitate an interaction of CheV D238E with PctA_CF.

To assess whether CheV D238E also interacts with the cytosolic fragments of other CheV-dependent chemoreceptors, we titrated the cytosolic fragments of McpK (CheV-dependent) and CtpM (CheV-independent) with CheV D238E. We observed binding of CheV D238E to McpK_CF, but not to CtpM_CF (Fig. S7). Although the heats generated by the interaction with McpK_CF were inferior to those of McpN_CF (Fig. 5), a similar biphasic binding curve was obtained and dissociation constants were in the nanomolar range. The differential interaction of CheV D238E thus agrees with the failure of the *cheV* mutant to respond to nitrate and α-ketoglutarate while retaining responses mediated by PctA (L-Val chemotaxis) and CtpM (L-malate chemotaxis) (Fig. 3A). Our data suggest that phosphorylation of CheV is required to interact with chemoreceptors and confirm the functional relevance of CheV phosphorylation.

## DISCUSSION

The hexagonal chemosensory array is formed by chemoreceptors, the CheA autokinase, and at least one coupling protein. CheW is a coupling protein in all species, but many species also have CheV. The composition of these arrays is highly variable among bacteria (57) and may include additional proteins, as in *Vibrio cholera* (58, 59). The coupling proteins are essential for the activity of chemosensory pathways. However, the importance of CheV is poorly understood, despite its being present in ~60% of prokaryotes with chemotaxis systems (19). Also, the functional consequences of CheV phosphorylation have been unclear. However, it is noteworthy that CheV is typically present in bacteria that have many different chemoreceptors (19).

Previous studies have shown that: (ⅰ) CheV increases the kinase activity of CheA (14); (ⅱ) CheV is phosphorylated by CheA (18, 60); (ⅲ) CheW and CheV integrate similarly into the chemoreceptor baseplate, indicating that both play a role in establishing the array structure (57, 61); (ⅳ) the interaction between CheV and chemoreceptors occurs through the CheW-like domain of CheV (11); and (ⅴ) CheV has co-evolved with certain chemoreceptors (19). We show here that, in *P. aeruginosa*: (ⅰ) CheV participates in signaling through the McpN, McpK, TlpQ, PctD, and Aer chemoreceptors (Fig. 3A and B); (ⅱ) both CheV and CheW are essential for signaling through these five chemoreceptors (Fig. 3 and 4; Fig. S4); (ⅲ) the non-phosphorylatable D238S variant of CheV is inactive (Fig. 3C); (ⅳ) unphosphorylated CheV fails to bind to the cytosolic fragments of chemoreceptors (Fig. 5); and (ⅴ) the phosphorylation mimic D238E variant of CheV binds with high affinity to the cytosolic fragments of McpN and McpK, but not to the cytosolic fragment of PctA and CtpM (Fig. 5; Fig. S7).

These results suggest that CheV is a regulatory protein that modulates signaling through specific chemoreceptors. Bacteria encoding CheV proteins have, on average, about five times more chemoreceptors than those without CheVs (19). Thus, CheV may facilitate the coordination of chemotaxis responses in complex, multi-chemoreceptor systems. Our previous proteomics studies have quantified the levels of chemosensory proteins in *P. aeruginosa* PAO1 under three growth conditions (62, 63). Under all growth conditions, we observed a two to four-fold excess of CheW_1_ over CheV, reinforcing the notion that CheV acts only on a subset of chemoreceptors.

Our data indicate that phosphorylation of CheV is required for interaction with McpN and McpK and potentially for the formation of the signaling complex. We hypothesize that the phosphorylation state of CheV may determine the magnitude of signaling through CheV-dependent receptors.

We show that the inactivation of *cheV* significantly decreased chemotaxis to nitrate, histamine, α-ketoglutarate, acetylcholine, and oxygen, whereas responses to L-malic acid, inorganic phosphate, L-Val, L-Gln, and γ-aminobutyrate were unaffected (Fig. 3A). The responses to nitrate, α-ketoglutarate, and acetylcholine are each mediated by a single chemoreceptor: McpN (33), McpK (37), and PctD (44), respectively. Receptors that were insensitive to the presence of CheV were the malate receptor CtpM (34), the phosphate-specific CtpL and CtpH receptors (38), and the three paralogous amino acid receptors PctA, PctB, and PctC (41). Histamine chemotaxis was decreased but not eliminated in the absence of CheV. TlpQ is the primary receptor for histamine, but two other chemoreceptors also mediate histamine chemotaxis (36). If one or two of these receptors do not require CheV, this partial inhibition is explained.

What features distinguish CheV-dependent from CheV-independent receptors? We have clustered the sequences of all 23 transmembrane chemoreceptors of *P. aeruginosa* to determine whether the domains of CheV-dependent and CheV-independent chemoreceptors may form different clusters. However, we were unable to detect any obvious correlation between the sequence clustering and receptor dependence on CheV (Fig. S8). A number of studies have defined the CheW-binding site on chemoreceptors (64–68). Although the binding site for CheV has not been rigorously determined, some data suggest that it binds to the same site as CheW (7, 13, 19). A sequence alignment of the signaling domains from the 11 chemoreceptors analyzed in this study reveals a high conservation of amino acids within the CheW-binding site across both protein families (Fig. S9). The only variation observed among the different receptors lies in a single amino acid at position 574 (TlpQ numbering), where either aspartic acid (D) or glutamic acid (E) is present. Notably, the specific amino acid at this position is not conserved between CheV-dependent and CheV-independent chemoreceptors (Fig. S9). Because CheV co-evolved with a particular chemoreceptor family, the CheW-binding site and phospho-CheV binding site are unlikely to be identical (19). The critical differences may lie in regions located rather far from the known CheW-binding site.

A major gap in our knowledge about chemosensory pathways is in understanding to what degree, in bacteria with multiple chemosensory systems, these pathways cross-talk with each other and other signaling networks. Whereas some reports show that these pathways are insulated (30, 69, 70), other studies show that they communicate with other systems (71–73). The question of insularity is related to the degree of specificity with which the different signaling proteins assemble to pathways. The *cheW*_1_ mutant is largely impaired in chemotaxis (Fig. 2), indicating that the remaining CheW homologs are unable to participate in this pathway. The interaction of CheW homologs only with their corresponding pathways is consistent with previous findings showing specific signaling protein interaction (30, 49). Our demonstration that a single CheW is needed for chemotaxis in *P. aeruginosa* is similar to the situation in *V. cholerae* and *Rhodobacter sphaeroides*. They possess two or three chemotaxis signaling pathways, respectively (9), but use only a single CheW for chemotaxis (74, 75). However, the spirochete *Borrelia burgdorferi* requires two of its three CheW proteins for chemotaxis (76).

CheV was first discovered in *B. subtilis*. Initial studies suggested redundancy between CheV and CheW, as mutants deleted for either gene maintained chemotaxis, whereas the double mutant was non-chemotactic (10). A similar redundancy has been observed in *C. jejuni* (11). In contrast, our results show that CheV and CheW_1_ are non-redundant; mutants in either the *cheV* or *cheW*_1_ gene are defective in nitrate and α-ketoglutarate chemotaxis (Fig. 4). Our findings are reminiscent of those made with *H. pylori*, in which the absence of *cheW* completely abolishes chemotaxis and the absence of *cheV_1_* severely compromises chemotaxis (12–14). Our work provides the insight that CheV can interact with a subset of receptors to provide a mechanism for maintaining segregated control of their chemosensory signaling.

## MATERIALS AND METHODS

Strains, plasmids, oligonucleotides, and culture conditions: Bacteria and plasmids used in this study are described in Table S2. *P. aeruginosa* strains were grown routinely at 30°C and 37°C, respectively, in lysogeny broth (LB) or M9 minimal medium supplemented with 6 mg/L Fe-citrate, trace elements (77), and 15 mM glucose as carbon source. *E. coli* strains were grown at 37°C in LB. *E. coli* DH5α was used as a host for gene cloning. When appropriate, antibiotics were used at the following final concentrations (in μg/mL): ampicillin, 100; kanamycin, 50; streptomycin, 50 (*E. coli*) and 100 (*P. aeruginosa* strains); gentamicin, 10 (*E. coli* strains) and 50 (*P. aeruginosa* strains); tetracycline, 60; rifampin, 10; chloramphenicol, 25. Sucrose was added to a final concentration of 10% (w/v) when required to select derivatives that had undergone a second crossover event during marker-exchange mutagenesis.

*In vitro* nucleic acid techniques: Total DNA extraction was performed using the Wizard genomic DNA purification kit (Promega). Plasmid DNA was isolated using the NZY-Miniprep kit (NZY-Tech). For DNA digestion, alkaline phosphatase, and ligation reactions, manufacturers’ instructions were followed (New England Biolabs and Roche). Competent cells were prepared using calcium chloride (78). Transformations and electroporations were performed following standard protocols (78). DNA fragments were recovered from agarose gels using the Qiagen gel extraction kit. PCRs were purified using the Qiagen PCR Clean-up kit. Phusion high-fidelity DNA polymerase (Thermo Fisher Scientific) was used in the amplification of PCR fragments for cloning. Sequences of these PCR fragments were verified by DNA sequencing.

Construction of plasmids: For the construction of plasmids for protein overexpression and gene complementation, DNA sequences were amplified by PCR and cloned into pET28b(+) or pBBR-based plasmids, respectively. To generate pCheV_Paer_D238S and pET28b-CheV-D238E, the phosphorylatable aspartate (D238) of PA3349 (CheV) was replaced by serine or glutamic acid by overlapping PCR. Complementation plasmids were transformed into *P. aeruginosa* strains by electroporation.

Chemotaxis assays: Overnight cultures in M9 minimal medium supplemented with 6 mg/L Fe-citrate, trace elements (77), and 15 mM glucose were used to inoculate fresh medium to an OD_660_ of 0.05. Cells were cultured at 37°C (*P. aeruginosa*) to an OD_660_ of 0.4–0.5. Subsequently, cells were washed twice by centrifugation (1,667 ×* g* for 5 min at room temperature) and resuspension in chemotaxis buffer (50 mM KH_2_PO_4_/K_2_HPO_4_, 20 mM EDTA, 0.05% [v/v] glycerol, pH 7.0). Cells were then resuspended in the same buffer at an OD_660_ of 0.1, and 230 µL aliquots of the resulting cell suspension were placed into the wells of 96-well microtiter plates. Then, 1 µL capillaries (Microcaps, Drummond Scientific) were heat-sealed at one end and filled with buffer (control) or chemoeffector solution prepared in chemotaxis buffer. The capillaries were rinsed with sterile water and immersed into the bacterial suspensions at their open ends. After 30 min, capillaries were removed from the wells, rinsed with sterile water, and emptied into 1 mL of chemotaxis buffer. Serial dilutions were plated onto M9 minimal medium plates supplemented with 15 mM glucose and incubated at 30°C prior to colony counting. For each strain, the number of bacteria that swam into buffer-containing capillaries (representing bacterial random movement) was subtracted from the number of cells that swam into ligand-containing capillaries (representing directed and random motility). The resulting data are plotted. Data are the means and standard deviations of at least three biological replicates conducted in triplicate.

Aerotaxis assays: These assays were carried out using the tube test method as described previously (79) with some modifications. Briefly, *P. aeruginosa* strains were grown in MS medium (30 mM Na_2_HPO_4_, 20 mM KH_2_PO_4_, 25 mM NH_4_NO_3_, 1 mM MgSO_4_) supplemented with 6 mg/L Fe-citrate, trace elements (77) and 15 mM glucose. Fresh medium was inoculated to an OD_660_ of 0.05. At an OD_660_ of 0.4, cells were washed twice with chemotaxis buffer (50 mM KH_2_PO_4_/K_2_HPO_4_, 20 mM EDTA, 0.05% [v/v] glycerol, pH 7.0) and concentrated to an OD_660_ of 0.5 in the same buffer. Subsequently, 1.5 mL of cell suspensions were mixed with the same volume of 0.5% (w/v) bacto-agar (Difco) prepared in chemotaxis buffer. This mixture was poured into sterile glass tubes, and pictures were taken after 1 h incubation at 30°C. Aerotactic responses are visualized by the formation of a clear band below the agarose/air interface.

Protein overexpression and purification: *E. coli* BL21(DE3) harboring pET28b-derived expression plasmids were grown in 2 L Erlenmeyer flasks containing 500 mL LB medium supplemented with kanamycin. Cultures were grown under continuous stirring (200 rpm) at 30°C. At an OD_660_ of 0.5–0.6, protein expression was induced by the addition of 0.1 mM isopropyl-β-D-thiogalactopyranoside. Growth was continued at 18°C overnight, and cells were harvested by centrifugation at 10,000 ×* g* for 20 min at 4°C. Proteins were purified by metal affinity chromatography. Cell pellets from a 1 L culture were resuspended in 40 mL of buffer A (30 mM Tris/HCL, 300 mM NaCl, 5% (v/v) glycerol, 10 mM imidazole, 0.1 mM EDTA, 5 mM 2-mercaptoethanol, pH 8.5) containing 1 mM PMSF protease inhibitor (Thermo Fisher Scientific) and Benzonase (Merck). Cells were then broken by French press treatment at a gauge pressure of 1000 lb/in^2^. After centrifugation at 20,000 ×* g* for 1 h, the supernatants were loaded onto a 5 mL HisTrap column (Amersham Bioscience) equilibrated in buffer A. After a wash with 40 mL of buffer A containing 40 mM imidazole, proteins were eluted by a linear gradient of 40 to 500 mM imidazole in buffer A.

ITC: Titrations were performed on a VP microcalorimeter (MicroCal, Northampton, MA, USA) at 25°C. Freshly purified proteins were dialyzed into 3 mM Tris, 3 mM PIPES, 3 mM MES, 5 mM 2-mercaptoethanol, pH 8.0. Ten µM solutions of either McpN_CF, PctA_CF, McpK_CF, or CtpM_CF were placed into the calorimeter sample cell and titrated at 240-s intervals with 12.8 µL aliquots of 113 µM CheV or CheV D238E. To assess the role of CheW_1_ in binding, the protein was added to a final concentration of 113 µM to PctA_CF and CheV D238E. The PctA_CF/CheW_1_ mixture was placed into the sample cell and titrated with the CheV D238E/CheW_1_ mixture. To correct for reactant dilution, the average enthalpy of the final peaks observed after saturation was subtracted from the titration data. The data were normalized to the ligand concentrations, and the first data point was removed. Data were analyzed using the "Two Binding Sites" model in the MicroCal version of Origin software for ITC.

## Data Availability

The data sets used and/or analyzed during the current study are available from the corresponding author on reasonable request.

## SUPPLEMENTAL MATERIAL

Supplemental Material mbio.02874-25-s0001.docx Supplemental figures and tables.

ASM does not own the copyrights to Supplemental Material that may be linked to, or accessed through, an article. The authors have granted ASM a non-exclusive, world-wide license to publish the Supplemental Material files. Please contact the corresponding author directly for reuse.

## Note

The footnotes in Tables are very small and hardly visible.
