# Supplementary material for: Phospho-mimetic CheV interacts with a subset of chemoreceptors
Source: mBio. 2025 Nov 10;16(12):e02874-25. doi: 10.1128/mbio.02874-25 (PMC12691637; doi:10.1128/mbio.02874-25)
Supplement: Supplemental Material — Supplemental figures and tables. [file mbio.02874-25-s0001.docx]

**Supplementary Information**

To

**Phospho-mimetic CheV interacts with a subset of chemoreceptors**

By

Miguel A. Matilla, Mario Cano-Muñoz, Elizabet Monteagudo-Cascales, Tino Krell

**Supplementary Figures**

**Fig. S1) Sequence alignment of the CheW homologs of *Pseudomonas aeruginosa* PAO1**. Sequences were aligned with the CLUSTALW algorithm of the npsa suite (1) using the GONNET weight matrix, a gap opening penalty of 10 and a gap extension penalty of 0.2. Red: identical; green: highly similar; blue: weakly similar.

10 20 30 40 50 60

| | | | | |

CheW2 MNGTE-------------------------------------------------------

ChpC MNQAV-----------------IEQDGMSRN----T------------------------

WspD MVEASLTLLDTQQVDDCWNRIGVHGDKSCERLAEHVHCRNCEVYAAAATYLLDRIALRQD

PilI MSDVQTPF-----------QLLVDIDQRCRRLAAGL------------------------

WspB MTVSR-------------------------------------------------------

CheW1 MSKAT-------------------------------------------------------

70 80 90 100 110 120

| | | | | |

CheW2 ------------ASQA-NGPAQEYLTFTLGREEYAIDILRVQEIRGYDQVTAIANAPP-F

ChpC ------------PANP-TPDSLTGLLLPLSDRTLLLPNVAVAELIAYRNPQVAAGLPQ-W

WspD QLDSAETMDSQREQSD-LGETRSILVFRLGEEWFGLATGSLVEVAPMNPIHSLPHQRSRA

PilI -----------PAQQE-AVQSWSGIGFRMGGRFFVAPMGEVGEVLHEPRYTQLPGVKT-W

WspB ------------STGDHRRTSKLFLLFRMEGDRYALDAREVVEVLPLLRLKRIPEAPE-W

CheW1 ------------AQSA-EDPILQWVTFRLDNESYGINVMQVQEVLRYTEIAPVPGAPS-Y

130 140 150 160 170 180

| | | | | |

CheW2 IKGVINLRGAIVPIVDLRIKFHLAE--VT-YDPF---TVVIILNIGR------RIVGVVV

ChpC YLGQVAWRDLRLPLLSFEAASSGEQ--QPVLGSS---ARVVVINALGGRP-HVKFLALLV

WspD LQGVTNVRGALVACLSLGELLDLEPGAAP-VSERRVVPRMLIISAAG------GPVVAPV

PilI VKGVANVRGRLLPIMDLCGFLGTEL--SP-LRKQ---RRVLVVEHLD------VFAGLIV

WspB VAGVFSHRGVLVPVLDLCAMAFGRA--AL-ARTS---TRIVLVEYRARQDREPVWLGLIL

CheW1 VLGIINLRGNVVTVIDTRQRFGLDP--AP-VSDN---TRIVIIEADK------QVVGILV

190 200 210 220 230 240

| | | | | |

CheW2 DSVSDVIALGGDAIRP-PPEFGASF-DTEYLLGLATAGERMLILVDIERLMTSREMA---

ChpC QGIPRSVRLDANLAGTAAPLVALEL-AAVDIGGET------ARIPDLAGL--EEK-----

WspD EEVDGIHAIPLARILP-PNHADGQA-SRRHVAGVLQWRERSITLLDEGPLLQA-------

PilI DEVFGMQHFPVDTFSEQLPPLEA-A-LQPFIHGVFHREQPWLV-FSPHALAQHQG-----

WspB EQATDTLRCEPSAFRD----YGLDNGGARYLGPVYEGPRGLVQWVRVEALLPDEVRALLF

CheW1 DSVAEVVYLKQSEIET-APNVGNEE-SAKFIQGVCNKNGELLILVELDKMMTEEE-----

CheW2 --LVDEAAA

ChpC --LADAGLI

WspD ---VARSLA

PilI ---FLDVAV

WspB PPECGEGTA

CheW1 --WSELGSI

**Fig. S2) Quantitative capillary chemotaxis assays of *Pseudomonas aeruginosa* PAO1 to histamine and acetylcholine (A) and aerotaxis (B) assays, a mutant in *cheV* harbouring the empty expression plasmid, and the *cheV* mutant harbouring a plasmid encoding *cheV*.** For the chemotaxis assays, data were corrected for the number of bacteria that swam into buffer-containing capillaries (8,073 ± 719 for PAO1 (pBBR1MCS2_START); 7,487 ± 841 for *cheV*::tn (pBBR1MCS2_START); 7,900 ± 2,404 for *cheV*::tn (pCheV_Paer). pBBR1MCS2_START: empty plasmid; pCheV_Paer: pBBR1MCS2_START plasmid encoding wt CheV. For each chemoeffector, data with different letters are statistically different, Student’s t-test: P-value <0.05.

**

**

**Fig. S3) Superimposition of the AlphaFold model of the receiver domain of *Pseudomonas aeruginosa* PAO1 CheV (cyan) with the three-dimensional structure of the phosphorylated form of the Spo0A response regulator (green, pdb ID 1QMP).** A) side view B) top view. The phosphorylated Spo0A aspartate 57 and its equivalent D238 in CheV are shown in stick mode.

**
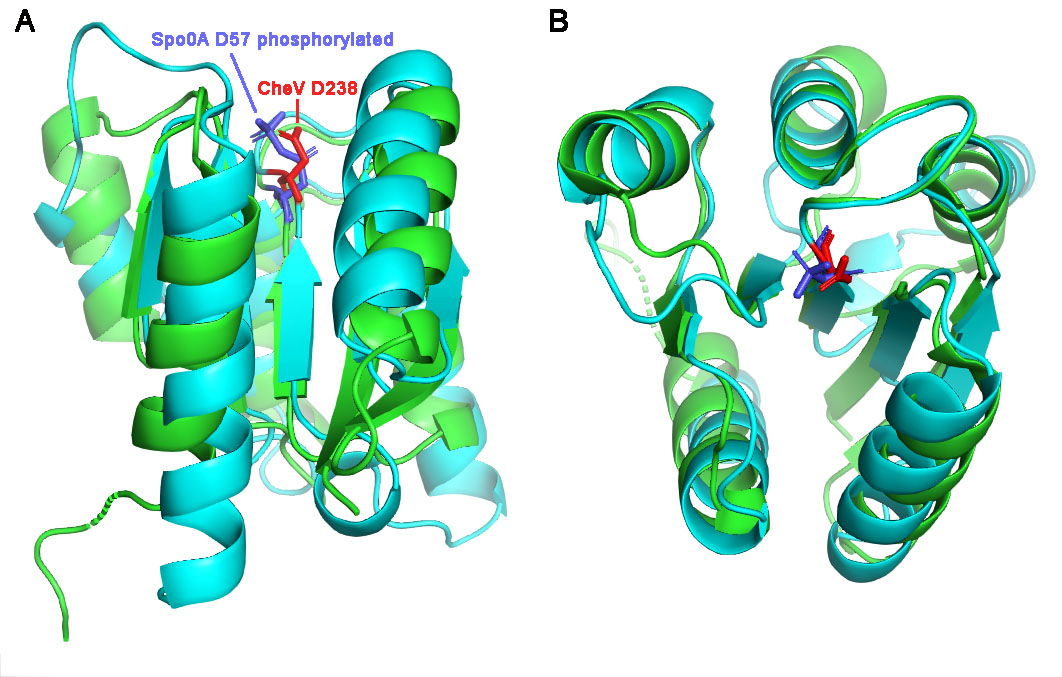
**

**Fig. S4) Quantitative capillary chemotaxis assay of the *Pseudomonas aeruginosa* PAO1 and a mutant in *cheW*_1_ to 5 mM histamine or 0.01 mM acetylcholine**. Data were corrected for the number of bacteria that swam into buffer-containing capillaries (4,273 ± 313 for wt; 3,329 ± 773 for *cheW*_1_::tn).

**
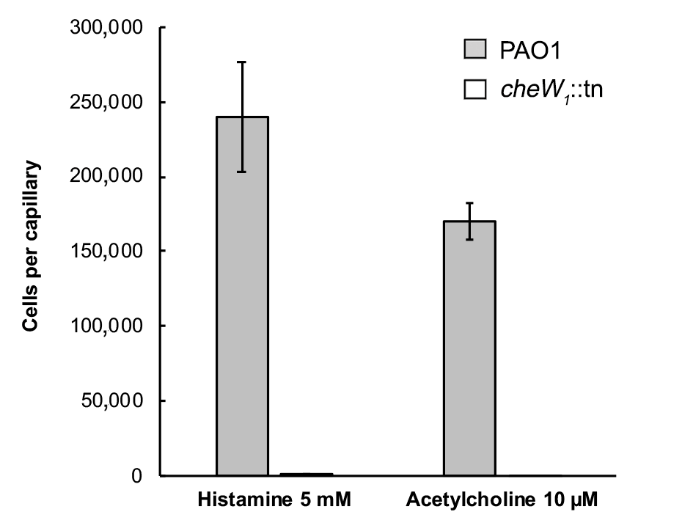
**

**Fig. S5) Hypothetical model of interaction of CheV D238E with the McpN signaling domain.** This model is based on the isothermal titration calorimetry data shown in Fig. 5. Alphafold2 models of the dimeric McpN signaling domain and CheV D238E were generated and used for a structural alignment with the ternary complex chemoreceptor/CheW/CheA (domains P4 and P5) from *Thermotoga maritima* (PDB ID: 3UR1)(2). ITC data showed two binding events which are hypothesized to correspond to the sequential binding of two CheV to either side of the dimeric McpN signaling domain. The *K*_D_ values obtained by ITC are annotated.

**
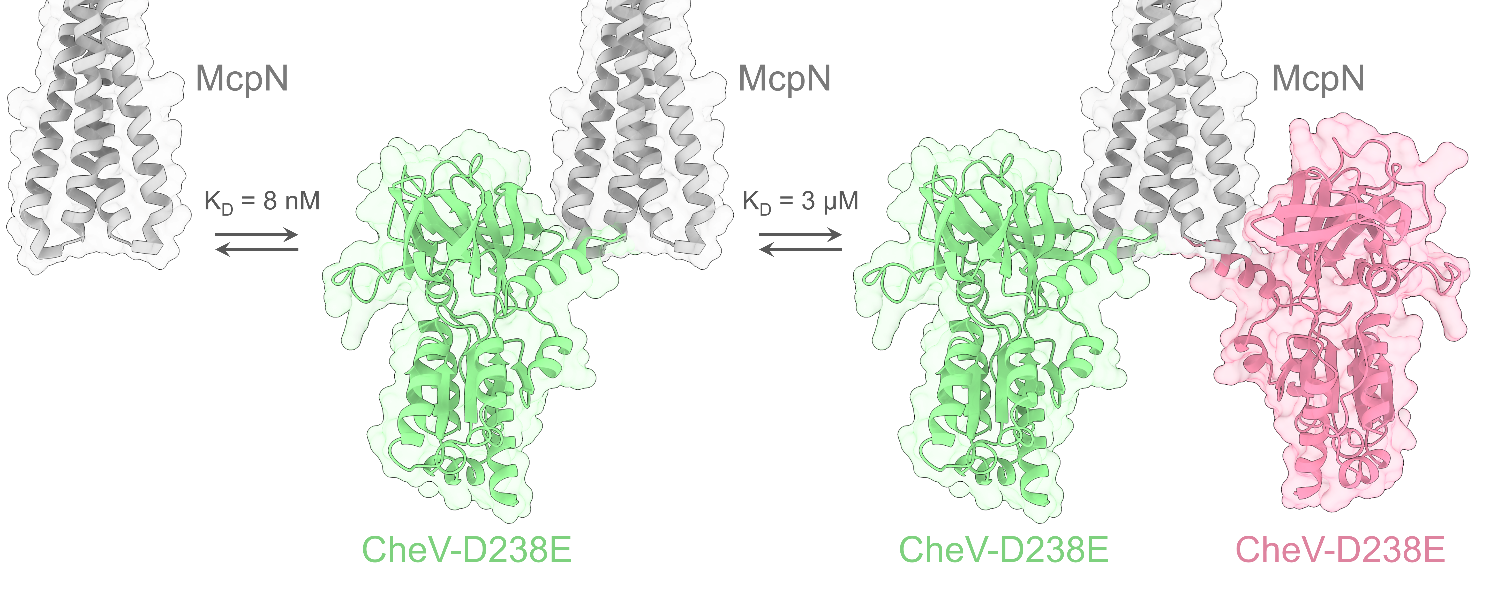
**

**Fig. S6) Assessment of the role of CheW_1_ in the potential binding of CheV D238E to the cytosolic fragment of the PctA chemoreceptor.** Microcalorimetric titration of a mixture of 113 µM CheW_1_ + 10 µM PctA_CF (in the sample cell) with a mixture of 113 µM CheW_1_ + 113 µM CheV D238E (in the injector syringe). A single 1.60315 µl injection was followed by a series of 12.8252 µl injections. Peaks are small and uniform representing dilution heats.

**

**

**Fig. S7) Microcalorimetric titrations of the cytosolic fragment of the McpK (A) and CtpM (B) with CheV D238E.** McpK and CtpM are CheV-dependent and CheV-independent receptors, respectively. In both experiments 10 µM solutions of receptor cytosolic fragments were titrated with 113 µM CheV D238E. A single 1.60315 µl injection was followed by a series of 12.8252 µl injections. Data were analysed with the Sequential Binding Site model of the MicroCal version of ORIGIN and resulting parameters are indicated.

**

**

**Fig. S8) Sequence clustering of the cytosolic fragments of *P. aeruginosa* PAO1 transmembrane chemoreceptors.** Chemoreceptors that were sensitive to the action of CheV are shown in red, and those that were insensitive in blue (Fig. 3A). The cytosolic chemoreceptors PA1423 (BdlA), PA1930 (McpS) and PA0176 (McpB/Aer2) have not been included in this analysis. Analysis carried out using TREND and default settings (3).

**
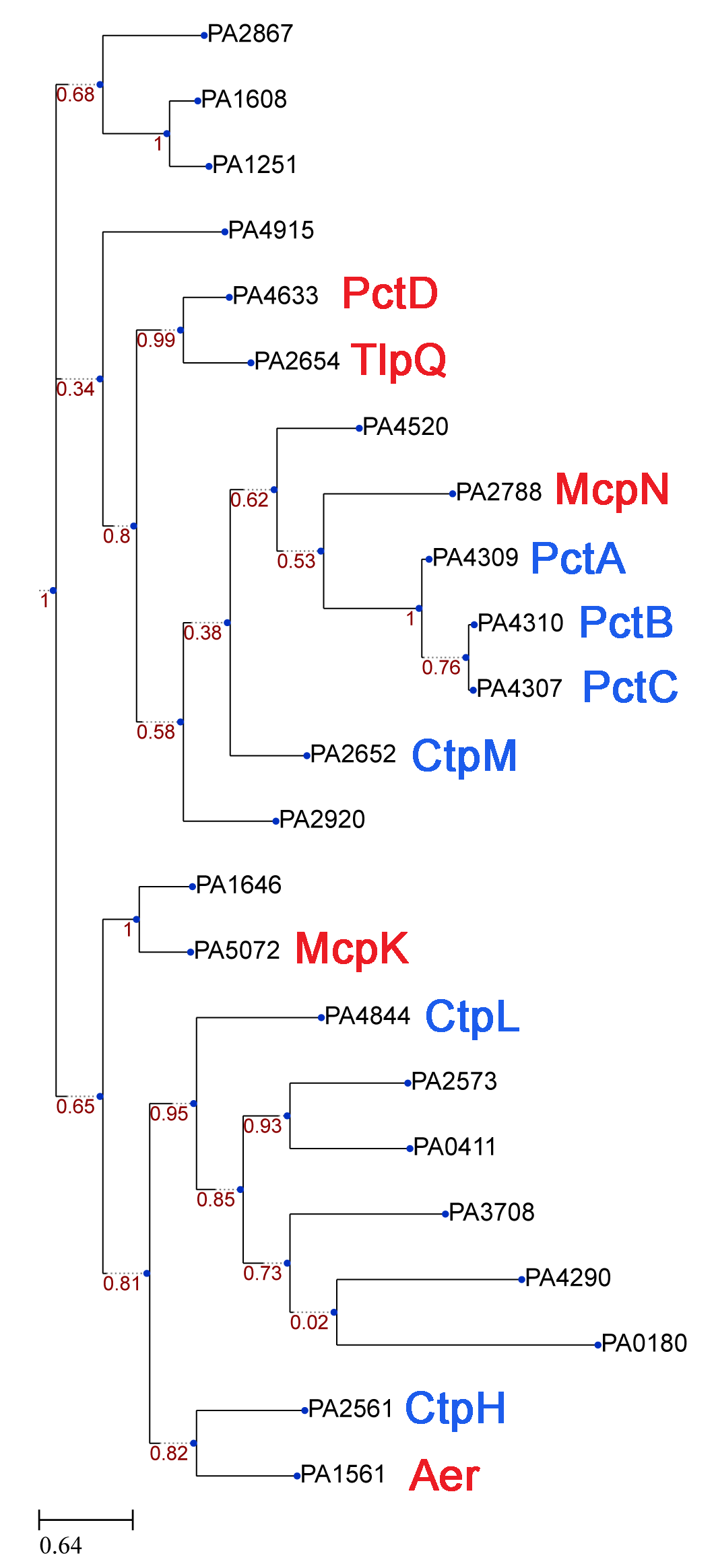
**

**Fig. S9) Sequence alignment of the cytosolic fragments of the 11 chemoreceptors studied.** Sequences were aligned with the CLUSTALW algorithm of the npsa suite (1) using the GONNET weight matrix, a gap opening penalty of 10 and a gap extension penalty of 0.2. Red: identical; green: highly similar; blue: weakly similar. The positions of amino acids that were previously shown to interact with CheW (4–6) are shaded in yellow. TlpQ numbering is shown. CheV-dependent receptors are in bold.

390 400 410 420 430 440

| | | | | |

**TlpQ** ------ARGVTRPILGVAHMLRDIASGEGD-LTQR----LPHT-G-RDELGELAGWFNRFLDKLQPIIRDVKVSVRDARS

CtpH -----VNRNLVRPVQRLIEHIAQL--SHGD-FGER----IEIR-R-KDELGKLALAANTLRDFLVDIFDRLRRSTRDLDS

**PctD** ---------IARPLRQLVGMLDDIAQGEGD-LTRR----LSSE-R-ADELGSIAKGFNTFLGKLQNMIGQVVQSVQKVSD

PctC ---------LMQPLTDMGRAMQDIAQGEGD-LTKR----LKVT-S-NDEFGTLANAFNRFVERIHESIREVAGTARQLHD

**McpK** VAGWAINRQIVRPLDEALAQAEAI--AAGD-LGKRPQNPLTLQ-R-RDELGQLQRVMQRMGDSLRELVGRISDGVSQLAS

PctB ----------MQPLTDMGRAMQDIAQGEGD-LTKR----LKVT-S-NDEFGALAISFNRFVERIHESIREVAGTARQLHD

CtpM -------NAMLRPLHQIRQNLDDIAAGEGD-LTRR----LPVT-S-YDELGELAGSFNRFVEKIHGLVRQIAGMTGDLKQ

CtpL DS---LQRRLARVLGQLVPALSAW--ADGD-FSRP----ISLRTR-TEDLRNLEDSLNRLRSFLAELVGAIHRRAEQVAG

**McpN** RQF--GLAPLMRQLRGLEVALTEV--GAAN-FTHA----LAAG-HADNEIGRIVAGYERMRQDVSGLLANVKRSAAETDK

**Aer/PA1561** ------LAGLRWQKRGLKRLMRLAEQTTSDPLIAQ----MYTD-S-RGDQARLEMAILSQDARLKTCLTRLQDTAEYLTE

PctA ----------MQPLHLMGRAMQDIAQGEGD-LTKR----LAVT-S-RDEFGVLGDAFNQFVERIHRSIREVAGTAHKLHD

450 460 470 480 490 500 510 520

| | | | | | | |

**TlpQ** TADQSAAISSQTSAGMQQQFREIDQVATASHEMTATAQDVARSAAQAADAARGADQATRDGLALIDRTTQSIDSLAANLT

CtpH ASGSLNAIASLMAAGTREQFSRTDQVATAMQEMSATAQEVARYAGDAARAADEADDSAQRGEDVMEETIRSIGEMRKEID

**PctD** SSEHTADIAIRTNQGVQQQLAEIELVATAVHEMTATAQDVARNATHAAEAANHADQAAHQGKQIVESSSAAIQALASEIG

PctC VAQLVVNASNSSMANSDEQSNRTNSVAAAINELGAAAQEIARNAADASHHASDANHQAEDGKQVVEQTIRAMNELSEKIS

**McpK** SAEELSAVTEQTRAGVNSQKVETDQVATAMHEMAATVQDVARNAELASQAARQADEEARQGDAVVDQAVTRIERLASEMD

PctB VAQLVVNASNSSMANSDEQSNRTNSVAAAINELGAAAQEIARNAADASHHASDANHQAEDGKQVVEQTIRAMNELSEKIS

CtpM LVEQMSAQAERSEQAMERQRHETDQVATAINEMSAAAHEVAQSAQRAAEAAQQTDHEGQAAKRVVDGSIERIHALVDEIR

CtpL SSQTLAEVSSGLHAGVERQAGDTGQIRDALGDMEAAIQQVAGDASQTADASRSAGQAVEHGQRVIGESLGGLRELVDEVQ

**McpN** DVAEALEQALGAGDQVARQHQDLDQVATAMNEMSATVAEVARHANHAAHSTRDAAALAHEGRRLVEHASSQTGALAEELE

**Aer/PA1561** QARQADTLAHHSSAGLEQQRAETEQVATAVNEMAATTQEVANNVQLTADATQKANELTSRGRDIAAETRNAIQRLSESVG

PctA VSQLVVNASNSSMANSDEQSNRTNSVAAAINELGAAAQEIARNAADASHHASDANHQAEDGKQVVEQTIRAMNELSEKIS

530 540 550 560 570 580 590 600

| | | | | | | |

**TlpQ** SAMGQVEQLASSSEEIGSVLEVIRAIAEQTNLLALNAAIEAARAGDAGRGFAVVADEVRNLARRTQDSVEQIRGVIEGLQ

CtpH HTVEVIRQLESDSGRIGKVLDVIRGIAEQTNLLALNAAIEAARAGDAGRGFAVVADEVRTLAQRTAESIAEIHQIIDTVQ

**PctD** RAVGVVQNLAKDSENINAILVAIRGIAEQTNLLALNAAIEAARAGEQGRGFAVVADEVRNLAQKTQQATEEIQSMIQQLQ

PctC ASCANIEALNSRTVNIGQILEVIKGISEQTNLLALNAAIEAARAGEAGRGFAVVADEVRNLAHRAQESAQQIQKMIEELQ

**McpK** VSSEAMARLKNESEQIGSVLDVIKSVAEQTNLLALNAAIEAARAGDAGRGFAVVADEVRGLAQRTQQSTAEIEGLIQRLQ

PctB ASCANIEALNSRTVNIGQILEVIKGISEQTNLLALNAAIEAARAGEAGRGFAVVADEVRNLAHRAQESAQQIQKMIEELQ

CtpM DSGTSLDSLQQDVQSIVSVLGVIRSIAEQTNLLALNAAIEAARAGEAGRGFAVVADEVRALASRTQQSTQEIQGMIDRLQ

CtpL GNAQSIERLAEESATIGSVLTVIRSIAEQTNLLALNAAIEAARAGDQGRGFAVVAEEVRSLAQRTAGATEEIQQLIGRLQ

**McpN** QTALALNTLHQHAGSVGQVLTVISSIAEQTNLLALNAAIEAARAGEAGRGFAVVADEVRSLANRTQQSTQEIQGLIEQLQ

**Aer/PA1561** ETGAAVSRLAQDSNEIGGVVDVIKGIADQTNLLALNAAIEAARAGDQGRGFAVVADEVRSLAQRTAASTEQIHHLIAKLQ

PctA ASCANIEALNSRTVNIGQILEVIKGISEQTNLLALNAAIEAARAGEAGRGFAVVADEVRNLAHRAQESAQQIQKMIEELQ

610 620 630 640 650 660 670 680

| | | | | | | |

**TlpQ** QGTRDVVDAMHGSHRQAQGSVEQVDEAVAALQRIGEAVTVINDMNLQIASAAEEQSSVAEEINRNVAAIRDVTESLSSQA

CtpH NGAVNAARAIESGQSRSEAGAEQVANAGAMLRQITASVESIRDMNRQIATAAEEQTAVAEEISRNLTEIASIASSNQEQV

**PctD** QGTRDVVKVMQDSQERTDDSVRHARQAAEALESITQAVSVINDMNTQIASAAEEQSAVAEDINRNVANIGQVANQVAGGA

PctC VGAREAVATMTESQRYSLESVEIANRAGERLGSVTSRIGEIDSMNQSVATATEEQTAVVDSLNMDITEINTLNQEGVENL

**McpK** QGAGEAAERLENSRSLTASTVELARRAGAALDSITRTVSDIQNMNLQIATAAEQQSTVAEEINRSVLSVRDVAEQSAAAS

PctB IGAQEAVSTMTESQRYSLESVEIANRAGERLSSVTGRIAEIDGMNQSVATATEEQTAVVDSLNMDITEINTLNQEGVENL

CtpM QGTNAAVDAMRRSGEAGEGTSNQANQAGDSLDAIAQLIATINAMNAQIASAAEEQTAVAEEINRSVHQIAGAVDSVADEA

CtpL QAARQSVEAMRSQVEHAERTAEQAGAAEGALDEVVAAIHTIGVMAERIAEGSTQQSQAVGEIRSHSERIHALGGENLRLI

**McpN** DGANDAVAAMRGSASHAQSNLVEADSAAQALGRIVATVEELDGLNQQIATAAEEQSQVAQDIDRNITNVSGLSEQAHEGT

**Aer/PA1561** NTANDAVHTMESGLQQAEAGVQRVLEADSALVGISEAVSNITEMTTQIAAAAEEQSAVAEEINRNISTIAALAEQTSDEA

PctA VGAREAVATMTESQRYSLESVEIANRAGESLSSVTRRIGEIDGMNQSVATATEEQTAVVDSLNMDITEINTLNQEGVENL

690 700 710

| | |

**TlpQ** EESAQVSQSLNRLANHQQGLMEQFKA

CtpH EQTEAASRDLHGLSAQLGDALQRLRA

**PctD** DEASQASAELTRLAEQQRRLVNQFRV

PctC QATLRACGELETQAGRLRHLVDSFKI

**McpK** EQTAASSGELARLGTQLQAQVGRFRL

PctB QATLRACGELETQAGRLRQLVDSFKI

CtpM QQGAQTARSLAQLGQGLGRLVGQFRI

CtpL GHSREQGEQLRQLGGDLRTTVQAFRL

**McpN**  AAVLSANQRVKEHMAGLRVVLGRFRT

**Aer/PA1561** LRTAKLSEELTTTAQSQYSLVERFNR

PctA QATLRACGELETQAGRLRQLVDSFKI

**Supplementary Tables**

**Table S1) Output from the analysis of CheV and CheV D238S by the Rosetta Stability and Scoring modelling software** (7)**.** The AlpaFold model of CheV was used. The D238S mutation was introduced into this model using the using YASARA Structure suit (https://www.yasara.org). CheV and CheV D238S were subjected to energy minimization and scoring under Rosetta’s full-atom energy function (8). For each variant, multiple independent minimizations were performed, and the best-scoring models were compared using total Rosetta energy units (REU). The change in Gibbs free energy (ΔΔG) corresponds to the difference between the ΔG score of the mutant and the wild-type protein. A negative ΔΔG score indicates an increase in stability. REU: Rosetta Energy Unit.

| Rosetta Score Term | CheV | CheV D238S | ΔΔG |
| --- | --- | --- | --- |
| **ΔG** | **-696.2 REU** | **-706.4 REU** | **-10.2 REU** |
| score | -696.2 | -706.4 |  |
| dslf_fa13 | 0 | 0 |  |
| fa_atr | -1716 | -1747 |  |
| fa_dun | 409.59 | 413.77 |  |
| fa_elec | -450.2 | -460.5 |  |
| fa_intra_rep | 2.65 | 2.641 |  |
| fa_intra_sol_xover4 | 54.101 | 53.987 |  |
| fa_rep | 147.25 | 153.95 |  |
| fa_sol | 1023.7 | 1036.7 |  |
| hbond_bb_sc | -31.07 | -31.53 |  |
| hbond_lr_bb | -74.29 | -74.1 |  |
| hbond_sc | -18.65 | -20.69 |  |
| hbond_sr_bb | -80.85 | -81.29 |  |
| linear_chainbreak | 0 | 0 |  |
| lk_ball_wtd | -35.15 | -33.06 |  |
| omega | 30.736 | 31.047 |  |
| overlap_chainbreak | 0 | 0 |  |
| p_aa_pp | -58.04 | -55.82 |  |
| pro_close | 5.919 | 5.907 |  |
| rama_prepro | -22.38 | -18.8 |  |
| ref | 116.76 | 118.62 |  |
| time | 0 | 0 |  |
| yhh_planarity | 0.004 | 0.004 |  |

**Table S2) Bacteria and plasmids used in this study.**

| **Bacterial strains** | **Genotype or relevant characteristics^a^** | **Reference/**  **source** |
| --- | --- | --- |
| **Bacteria** | | |
| *Escherichia coli* DH5α | *supE44 lacU169* (*Ø80lacZΔ M15*) *hsdR17* (r_K_^−^m_K_^−^) *recA1* *endA1* *gyrA96 thi-1* *relA1* | (9) |
| *E. coli* CC118λpir | *araD*, Δ(*ara*, *leu*), Δ*lacZ*74, *pho*A20, *galK*, *thi-1*, *rspE*, *rpoB*, *argE*, *recA1*, λ*pir* | (10) |
| *E. coli* BL21(DE3) | F^–^ *ompT* *gal* *dcm* *lon* *hsdS_B_*(*r_B_*^–^*m_B_*^–^) λ(DE3 [*lacI* *lacUV5*-*T7p07* *ind1* *sam7* *nin5*]) [*malB*^+^]_K-12_(λ^S^) | (11) |
| *E. coli* HB101 | F^-^ ∆*(gpt-proA)62 leuB6 supE44 ara-14 galK2 lacY1*Δ  *(mcrC-mrr) rpsL20* (Sm^R^) *xyl-5 mtl-1 recA13 thi-1* | (12) |
| *Pseudomonas aeruginosa* PAO1 | Wild type | (13) |
| *P. aeruginosa cheW*_1_::tn | PAO1 transposon mutant *pa1464*::IS*phoA*/hah; Tc^R^ | (14) |
| *P. aeruginosa cheW*_2_::tn | PAO1 transposon mutant *pa0177*::IS*phoA*/hah; Tc^R^ | (14) |
| *P. aeruginosa wspB*::tn | PAO1 transposon mutant *pa3707::*IS*lacZ*/hah; Tc^R^ | (14) |
| *P. aeruginosa wspD*::tn | PAO1 transposon mutant *pa3705::*IS*lacZ*/hah; Tc^R^ | (14) |
| *P. aeruginosa chpC*::tn | PAO1 transposon mutant *pa0415*::IS*lacZ*/hah; Tc^R^ | (14) |
| *P. aeruginosa cheV*::tn | PAO1 transposon mutant *pa3349*::IS*phoA*/hah; Tc^R^ | (14) |
| **Plasmids** | | |
| pET28b(+) | Km^R^; protein expression plasmid | Novogene |
| pBBR1MCS2_START | Km^R^; *oriRK2 mobRK2* | (15) |
| pCheV_Paer | Km^R^; a 1.0-kb PCR fragment containing the *cheV* (*pa3349*) gene from *P. aeruginosa* PAO1 cloned into the NdeI/EcoRI sites of pBBR1MCS2_START | This study |
| pCheV_Paer_D238S | Km^R^; a 1.0-kb PCR fragment containing the *cheV* (*pa3349*) gene from *P. aeruginosa* PAO1 with an aspartate-to-serine mutation at the phosphorylation site (D238S), cloned into the NdeI/EcoRI sites of pBBR1MCS2_START | This study |
| pET28b-CheV | Km^R^; pET28b(+) derivative containing a DNA fragment encoding CheV cloned into the NdeI/EcoRI sites | This study |
| pET28b-CheV-D238E | Km^R^; pET28b(+) derivative containing a DNA fragment encoding CheV (D238E) cloned into the NdeI/EcoRI sites | This study |
| pET28b_McpN_CF | Km^R^; pET28b(+) derivative containing a DNA fragment encoding the cytosolic fragment (CF) of the McpN chemoreceptor (amino acids 196 to 532) cloned into the NdeI/HindIII sites | GenScript |
| pET28b_PctA_CF | Km^R^; pET28b(+) derivative containing a DNA fragment encoding the cytosolic fragment (CF) of the PctA chemoreceptor (amino acids 301 to 629) cloned into the NdeI/XhoI sites | This study |
| pET28b_McpK_CF | Km^R^; pET28b(+) derivative containing a DNA fragment encoding the cytosolic fragment (CF) of the McpK chemoreceptor (amino acids 313-647) cloned into the NdeI/XhoI sites | This study |
| pET28b_CtpM_CF | Km^R^; pET28b(+) derivative containing a DNA fragment encoding the cytosolic fragment (CF) of the PctA chemoreceptor (amino acids 234-561) cloned into the NdeI/XhoI sites | This study |
| pET28_CheW_1_ | Km^R^; pET28b(+) derivative containing the *cheW*_1_ (*pa1464*) gene of *P. aeruginosa* PAO1 cloned into the NdeI/XhoI sites | This study |

### *^a^*Ap, ampicillin; Km, kanamycin; Sm, streptomycin; Cm, chloramphenicol; Gm, gentamicin

**References**

1. Combet C, Blanchet C, Geourjon C, Deleage G. 2000. NPS@: network protein sequence analysis. Trends Biochem Sci 25:147–50.

2. Briegel A, Li X, Bilwes AM, Hughes KT, Jensen GJ, Crane BR. 2012. Bacterial chemoreceptor arrays are hexagonally packed trimers of receptor dimers networked by rings of kinase and coupling proteins. Proc Natl Acad Sci U S A 109:3766–71.

3. Gumerov VM, Zhulin IB. 2020. TREND: a platform for exploring protein function in prokaryotes based on phylogenetic, domain architecture and gene neighborhood analyses. Nucleic Acids Res 48:W72–W76.

4. Li X, Fleetwood AD, Bayas C, Bilwes AM, Ortega DR, Falke JJ, Zhulin IB, Crane BR. 2013. The 3.2 Å resolution structure of a receptor: CheA:CheW signaling complex defines overlapping binding sites and key residue interactions within bacterial chemosensory arrays. Biochemistry 52:3852–3865.

5. Vu A, Wang X, Zhou H, Dahlquist FW. 2012. The receptor-CheW binding interface in bacterial chemotaxis. J Mol Biol 415:759–767.

6. Pedetta A, Parkinson JS, Studdert CA. 2014. Signalling-dependent interactions between the kinase-coupling protein CheW and chemoreceptors in living cells. Mol Microbiol 93:1144–1155.

7. Thieker DF, Maguire JB, Kudlacek ST, Leaver-Fay A, Lyskov S, Kuhlman B. 2022. Stabilizing proteins, simplified: A Rosetta-based webtool for predicting favorable mutations. Protein Sci 31:e4428.

8. Alford RF, Leaver-Fay A, Jeliazkov JR, O’Meara MJ, DiMaio FP, Park H, Shapovalov MV, Renfrew PD, Mulligan VK, Kappel K, Labonte JW, Pacella MS, Bonneau R, Bradley P, Dunbrack RL, Das R, Baker D, Kuhlman B, Kortemme T, Gray JJ. 2017. The Rosetta All-Atom Energy Function for Macromolecular Modeling and Design. J Chem Theory Comput 13:3031–3048.

9. Woodcock DM, Crowther PJ, Doherty J, Jefferson S, DeCruz E, Noyer-Weidner M, Smith SS, Michael MZ, Graham MW. 1989. Quantitative evaluation of *Escherichia coli* host strains for tolerance to cytosine methylation in plasmid and phage recombinants. Nucleic Acids Res 17:3469–3478.

10. Herrero M, de Lorenzo V, Timmis KN. 1990. Transposon vectors containing non-antibiotic resistance selection markers for cloning and stable chromosomal insertion of foreign genes in gram-negative bacteria. J Bacteriol 172:6557–6567.

11. Jeong H, Barbe V, Lee CH, Vallenet D, Yu DS, Choi SH, Couloux A, Lee SW, Yoon SH, Cattolico L, Hur CG, Park HS, Segurens B, Kim SC, Oh TK, Lenski RE, Studier FW, Daegelen P, Kim JF. 2009. Genome sequences of *Escherichia coli* B strains REL606 and BL21(DE3). J Mol Biol 394:644–652.

12. Boyer HW, Roulland-Dussoix D. 1969. A complementation analysis of the restriction and modification of DNA in *Escherichia coli*. J Mol Biol 41:459–472.

13. Stover CK, Pham XQ, Erwin AL, Mizoguchi SD, Warrener P, Hickey MJ, Brinkman FS, Hufnagle WO, Kowalik DJ, Lagrou M, Garber RL, Goltry L, Tolentino E, Westbrock-Wadman S, Yuan Y, Brody LL, Coulter SN, Folger KR, Kas A, Larbig K, Lim R, Smith K, Spencer D, Wong GK, Wu Z, Paulsen IT, Reizer J, Saier MH, Hancock RE, Lory S, Olson MV. 2000. Complete genome sequence of Pseudomonas aeruginosa PAO1, an opportunistic pathogen. Nature 406:959–964.

14. Jacobs MA, Alwood A, Thaipisuttikul I, Spencer D, Haugen E, Ernst S, Will O, Kaul R, Raymond C, Levy R, Chun-Rong L, Guenthner D, Bovee D, Olson MV, Manoil C. 2003. Comprehensive transposon mutant library of *Pseudomonas aeruginosa*. Proc Natl Acad Sci U S A 100:14339–14344.

15. Obranic S, Babic F, Maravic-Vlahovicek G. 2013. Improvement of pBBR1MCS plasmids, a very useful series of broad-host-range cloning vectors. Plasmid 70:263–267.
